# Lipoxin A_4_ Attenuates Cell Invasion by Inhibiting ROS/ERK/MMP Pathway in Pancreatic Cancer

**DOI:** 10.1155/2016/6815727

**Published:** 2015-11-16

**Authors:** Liang Zong, Jiahui Li, Xin Chen, Ke Chen, Wei Li, Xuqi Li, Lun Zhang, Wanxing Duan, Jianjun Lei, Qinhong Xu, Tao Shan, Qingyong Ma, Hao Sun

**Affiliations:** ^1^Department of Hepatobiliary Surgery, First Affiliated Hospital, Xi'an Jiaotong University, Xi'an 710061, China; ^2^Department of General Surgery, First Affiliated Hospital, Xi'an Jiaotong University, Xi'an 710061, China; ^3^Department of General Surgery, Second Affiliated Hospital, Xi'an Jiaotong University, Xi'an 710004, China

## Abstract

Lipoxin A_4_ (LXA_4_), an endogenous arachidonic acid metabolite, was previously considered an anti-inflammatory lipid mediator. But it also has the potential to inhibit cancer progression. To explore the therapeutic effect of LXA_4_ in pancreatic cancer, we used Panc-1 cells to investigate the mechanism by which LXA_4_ can attenuate pancreatic cancer cell invasion. Our data showed that LXA_4_ significantly inhibited both cell invasion and the expression of matrix metalloproteinase- (MMP-) 9 and MMP-2. Further experiments implied that LXA_4_ decreased the levels of intracellular reactive oxygen species (ROS) and the activity of the extracellular signal regulated kinases (ERK) pathway to achieve similar outcome to ROS scavenger N-acetyl-l-cysteine (NAC). However, a decreased level of intracellular ROS was not observed in cells treated with the specific ERK pathway inhibitor FR180204. The blocking of either intracellular ROS or ERK pathway caused the downregulation of MMP-9 and MMP-2 expression. Furthermore, tests revealed that LXA_4_ inhibited MMP-9 and MMP-2 at the mRNA, protein, and functional levels. Finally, LXA_4_ dramatically limited the invasion of CoCl_2_-mimic hypoxic cells and abrogated intracellular ROS levels, ERK activity, and MMPs expression. These results suggest that LXA_4_ attenuates cell invasion in pancreatic cancer by suppressing the ROS/ERK/MMPs pathway, which may be beneficial for preventing the invasion of pancreatic cancer.

## 1. Introduction

Pancreatic cancer is the fourth-leading cause of cancer-related death in the United States [1]. Although biochemical and clinical studies have led to significant advances, the five-year survival rate remains less than 7% [1]. High invasive and metastatic tendencies are important characteristics of pancreatic cancer, which partially result in rapid progression and poor prognosis. However, the mechanisms that lead to invasion and metastasis in pancreatic cancer are still poorly understood.

Lipoxin A_4_ (LXA_4_) is a type of metabolite that is derived from endogenous arachidonic acid (AA). Lipoxygenases (LOX), especially 5-LOX, 15-LOX, and 12-LOX, are key enzymes that contribute to LXA_4_ biosynthesis [2]. Interestingly, aspirin tends to acetylate cyclooxygenase-2 (COX-2), which changes its product from prostaglandin to an analogue of LXA_4_ or aspirin-triggered lipoxin (ATL) [2]. Previously, LXA_4_ was regarded as an anti-inflammatory, proresolution lipid that plays important roles in the programmed switch from inflammation to resolution [3, 4]. However, its various anticancer effects have been investigated in recent years. On the one hand, with its anti-inflammatory function, LXA_4_ may block carcinogenesis through the attenuation of chronic inflammation, which usually presents as premalignant lesions; on the other hand, cancer cell proliferation, apoptosis, migration [5], and angiogenesis [6] can also be influenced by LXA_4_ independent of its function in the resolution of inflammation.

Endogenous reactive oxygen species (ROS), including hydroxyl radical, superoxide anion, and hydrogen peroxide, are mainly produced on the mitochondrial inner membrane during the process of oxidative phosphorylation via the electron transport chain. Generally, ROS can be scavenged by antioxidant systems. However, in cancer cells, excessive ROS overwhelms the capacity of antioxidant systems, which leads to oxidative stress; this in turn has been demonstrated to promote cell migration, invasion, and metastasis [7, 8].

Matrix metalloproteinases (MMPs) are zinc-dependent endopeptidases that degrade extracellular matrix components. Specifically, MMP-9 and MMP-2 are thought to facilitate cancer invasion and metastasis. In pancreatic cancer, these two proteins are secreted by both pancreatic cancer cells and pancreatic stellate cells [9]. Our previous study demonstrated that miR-106a and miR-221/222 induced the overexpression of MMPs, which can significantly promote cell invasion [10, 11]. Additionally, the expression of MMPs is downregulated when the ROS/extracellular signal regulated kinases (ERK) pathway is blocked in breast [12] and prostate [13] cancers.

In this study, we demonstrate that LXA_4_ can effectively attenuate cell invasion and MMP-9/MMP-2 expression in pancreatic cancer by inhibition of intracellular ROS accumulation and ROS-induced ERK activation. Furthermore, LXA_4_ also reverses CoCl_2_ mimetic hypoxia-induced MMP-9/MMP-2 overexpression as well as cell invasion.

## 2. Materials and Methods

### 2.1. Materials

The reagents used in this study include 5(*S*), 6(*R*)-Lipoxin A_4_ (Cayman Chemical, Ann Arbor, MI, USA), N-acetyl-l-cysteine (NAC) (Sigma-Aldrich, MO, USA), and FR180204 (Sigma-Aldrich). The following antibodies were purchased from Bioworld (St. Louis Park, MN, USA): anti-MMP-9, anti-MMP-2, anti-ERK1/2, anti-phospho-ERK1/2; an anti-*β*-actin antibody was obtained from Sigma-Aldrich.

### 2.2. Cell Culture

The Panc-1 human pancreatic cancer-derived cell line was purchased from the Cell Bank of the Chinese Academy of Sciences (Shanghai, China). The cells were cultured at 37°C in 5% CO_2_ in Dulbecco's Modified Eagle's Medium (DMEM) (high glucose) (Gibco, Grand Island, NY, USA) supplemented with 10% heat-inactivated fetal bovine serum (FBS) (ExCell, South America) plus 100 U/mL penicillin and 100 *μ*g/mL streptomycin (Gibco).

### 2.3. Western Blot Analysis

Panc-1 cells cultured under each experimental condition were lysed in RIPA lysis buffer (50 mM Tris-HCl, pH 7.5, 150 mM NaCl, 1% Triton X-100, 2 mM EDTA, 1 mM sodium orthovanadate, 1 mM phenylmethanesulfonyl-fluoride, 10 *μ*g/mL aprotinin, and 10 *μ*g/mL leupeptin), proteinase inhibitors (Roche, Mannheim, Germany), and phosphatase inhibitors (Roche) on ice for 30 min. The extracts were centrifuged at 12,000 rpm for 20 min at 4°C. Total protein (100 *μ*g) was electrophoresed in a 10% SDS-PAGE gel and then transferred to PVDF membranes (Roche), which were then blocked with 10% nonfat dry milk in TBST (10 mM Tris-HCl, pH 8.0, 150 mM NaCl, and 0.05% Tween-20). The membranes were incubated with primary antibodies overnight at 4°C. After five washes of 10 min each in TBST, the membranes were incubated with HRP-conjugated secondary antibodies for 2 hours at 20°C and then washed again. The peroxidase reaction was performed using an enhanced chemiluminescence detection system to visualize the immunoreactive bands.

### 2.4. Cell Invasion Assay

A chamber-based cell invasion assay (Millipore, Billerica, USA) was performed to evaluate pancreatic cancer cell invasion. Briefly, the upper surface of the membrane was coated with Matrigel (BD Biosciences, Franklin Lakes, USA). Panc-1 cells (1 × 10^5^) were suspended in the upper chamber in FBS-free media and allowed to migrate down a serum gradient (10%) in the lower chamber. The medium was aspirated from the inside of the insert and the noninvasive cells on the upper side were removed by scraping with a cotton swab. The membrane was fixed in 4% paraformaldehyde and was stained with crystal violet. The number of invasive cells was counted in 10 random fields on each membrane and photographed at 200x magnification. The values reported here are averages of triplicate experiments.

### 2.5. Quantitative Real-Time RCR Assay (qRT-PCR)

Total RNA was extracted from Panc-1 cells with the Fastgen200 RNA isolation system (Fastgen, Shanghai, China), and reverse transcription was performed with a PrimeScript RT reagent Kit (TaKaRa, Dalian, China) according to the manufactures' instructions. Real-time PCR was conducted as previously reported [14]. The PCR primer sequences for MMP-9, MMP-2, and *β*-actin are shown in Supplemental Table  1 (in Supplementary Material available online at http://dx.doi.org/10.1155/2016/6815727). To quantitate the expression of each target gene, the expression was normalized to *β*-actin, and the comparative Ct method was used [15].

### 2.6. Assay of Intracellular ROS

The presence of intracellular ROS was tested as in a previous study. Panc-1 cells were incubated with 5 *μ*g/mL 2′-7′-dichlorofluorescein diacetate (DCF-DA) for 20 min. After washes with PBS, the cells were lysed in 1 mL RIPA buffer and were analyzed immediately by fluorimetric analysis at 510 nm. The data were normalized to the total protein content.

### 2.7. Enzyme-Linked Immunosorbent Assay (ELISA)

The cells were conditioned in serum-free medium for 24 h. The culture supernatants were collected and centrifuged at 1,500 rpm for 5 min to remove particles; the supernatants were frozen at −80°C until use. The MMP-9 and MMP-2 levels in the supernatants of Panc-1 cells were assessed using a commercially available ELISA kit (R&D Systems, USA) according to the manufacturer's recommendations.

### 2.8. Statistical Analysis

The data are presented as the mean ± the standard deviation (SD). The differences were evaluated by Student's *t*-test with SPSS 13.0. *P* values below 0.05 were considered statistically significant. All experiments were repeated independently at least three times.

## 3. Results

### 3.1. LXA_4_ Inhibits Cell Invasion and Decreases Expression of MMP-9 and MMP-2

**Figure 1:**
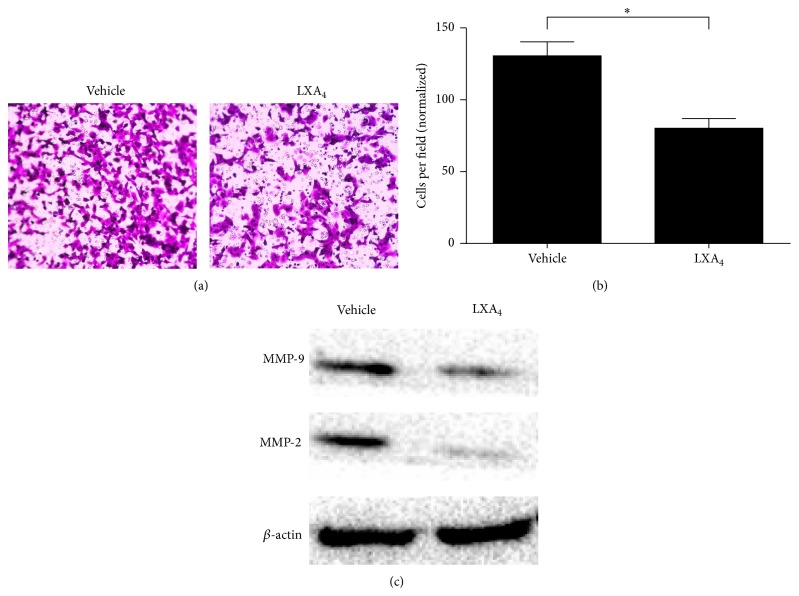
LXA_4_ inhibited cell invasion and decreased expression of MMP-9 and MMP-2. (a) Effect of LXA_4_ on cell invasion in Panc-1 cells. Cells were treated with either vehicle (methanol) or LXA_4_ (400 nM) and incubated for 24 hours. Then 1 × 10^5^ cells were transferred into transwell chambers covered with Matrigel. Cultured for 48 hours, cells were stained with 0.1% crystal violet and finally observed and counted under microscope. (b) The quantified results of (a). (c) Representative western blot analysis of MMP-9 and MMP-2 in cells treated like above.  ^*∗*^ *P* < 0.05 versus vehicle control.

To test the influence of LXA_4_ on pancreatic cancer* in vitro*, we chose the pancreatic cell line Panc-1, which was treated with either the vehicle control (methanol) or 400 nM LXA_4_ for 24 hours. Then, to test the invasive capability of the treated cells, a transwell assay was performed, which showed that 130.6 ± 9.7 cells in the vehicle control group passed through the Matrigel, whereas 80.2 ± 8.5 cells in the LXA_4_ group passed through the Matrigel (Figures 1(a) and 1(b)). This suggests that LXA_4_ could significantly suppress cell invasion. MMP-9 and MMP-2 are two widely accepted proteinases that facilitate cell invasion and metastasis. We also observed that compared with the vehicle control lower levels of MMP-9 and MMP-2 were expressed in Panc-1 cells after they were treated with LXA_4_ (Figure 1(c)).

### 3.2. LXA_4_ Attenuates Cell Invasion by Inhibiting ROS Pathway

**Figure 2:**
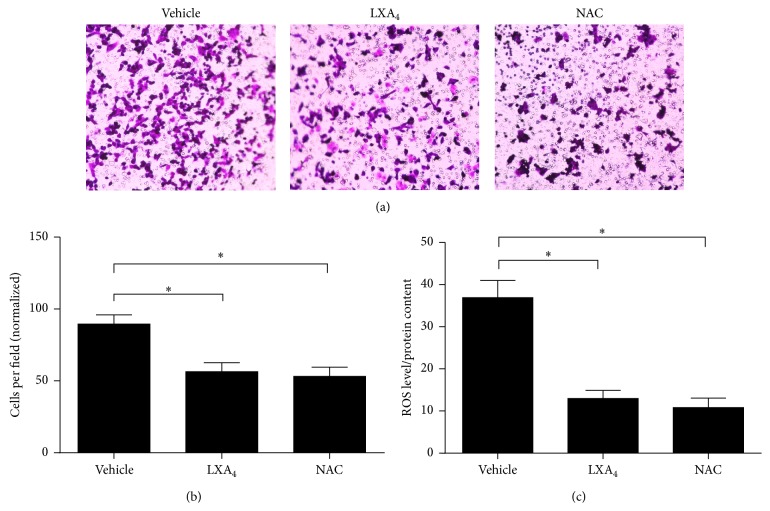
LXA_4_ attenuated cell invasion via inhibiting ROS pathway. (a) Cell invasion tested by transwell chamber in Panc-1 cells treated with vehicle (methanol), LXA_4_ (400 nM), or ROS scavenger NAC (20 mM). (b) The quantified results of (a). (c) Intracellular ROS determined in cells treated in (a). Cells incubated with DCF-DA for 20 min were washed with PBS three times and then lysed by RIPA lysis buffer and tested by fluorimetry at 510 nm. It was normalized by total protein.  ^*∗*^ *P* < 0.05 versus vehicle control.

It has been reported that elevated intracellular ROS tends to enhance cell invasion [16], whereas LXA_4_ can decrease intracellular ROS [17–19]. We treated Panc-1 cells with vehicle, LXA_4_, and ROS scavenger NAC at 20 mM. Then, we performed cell invasion assay, which demonstrated that fewer cells passed through the Matrigel after they were treated with LXA_4_ and NAC compared with cells that were treated with vehicle (Figures 2(a) and 2(b)). This demonstrated that ROS might be involved in the regulation of cell invasion. At the same time, based on the intracellular ROS levels that were detected in Panc-1 cells that were treated with vehicle, LXA_4_, and NAC, the data suggest that LXA_4_, similar to NAC, decreased the amount of intracellular ROS compared with the vehicle control (Figure 2(c)). These data supported the concept that the suppression of ROS pathway by LXA_4_ was responsible for attenuated cell invasion.

### 3.3. LXA_4_ Negatively Regulates Cell Invasion by Inhibiting ROS/ERK Pathway

**Figure 3:**
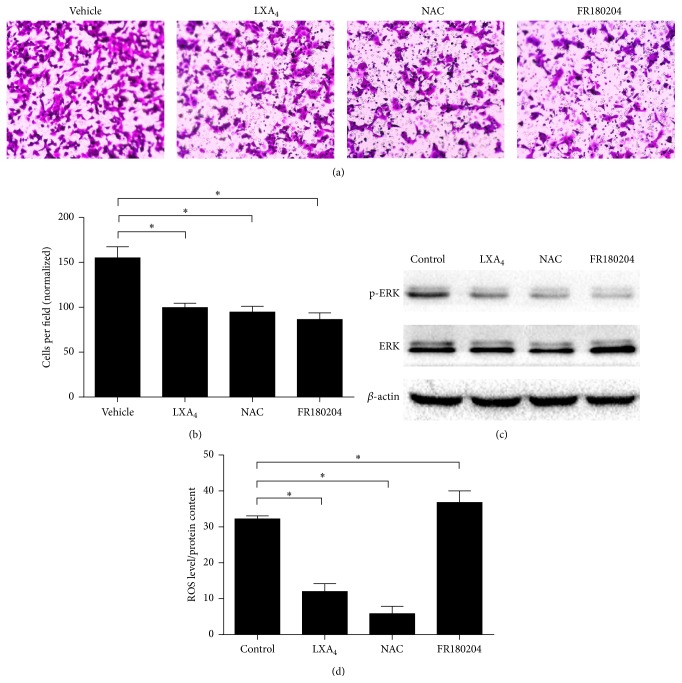
LXA_4_ negatively regulated cell invasion by inhibiting ROS/ERK pathway. (a) Influence of LXA_4_ on cell invasion in Panc-1 cells. Cells were treated with vehicle (methanol), LXA_4_ (400 nM), ROS scavenger NAC (20 mM), or ERK specific inhibitor FR180204 (10 *μ*M) for 24 hours. Then 1 × 10^5^ cells were transferred into transwell chambers covered with Matrigel. After forty-eight hours, cells were stained with 0.1% crystal violet, observed, and counted under microscope. (b) The quantified results of (a). (c) Representative western blot analysis of activated p-ERK and total ERK in cells treated as in (a). (d) Intracellular ROS determined in cells treated in (a). Cells incubated with DCF-DA for 20 min were washed with PBS three times and then lysed by RIPA lysis buffer and tested by fluorimetry at 510 nm. It was normalized by total protein.  ^*∗*^ *P* < 0.05 versus vehicle control.

The ERK pathway, which is overactive in pancreatic cancer, is widely accepted to affect cell invasion [20]. When exposed to the specific ERK pathway inhibitor FR180204 (10 *μ*M), cells present less aggressive invasion as LXA_4_ and NAC (Figures 3(a) and 3(b)), which suggests that ERK might mediate LXA_4_ attenuated cell invasion. Because ERK is reported to be a downstream pathway of ROS [12, 13], we detected ERK activity and showed phospho-ERK accounted for a lower proportion of total ERK when the cells were treated with LXA_4_ and NAC (Figure 3(c)). However, cells that were exposed to FR180204 failed to show a decrease in intracellular ROS (Figure 3(d)). Our data confirmed that ROS could induce ERK activation, which suggests that LXA_4_ could inactivate the ERK pathway via decreasing intracellular ROS. This in turn further downregulates cell invasion.

### 3.4. LXA_4_ Downregulated MMP-9/MMP-2 on Transcriptional Level rather than Translation or Secretion

**Figure 4:**
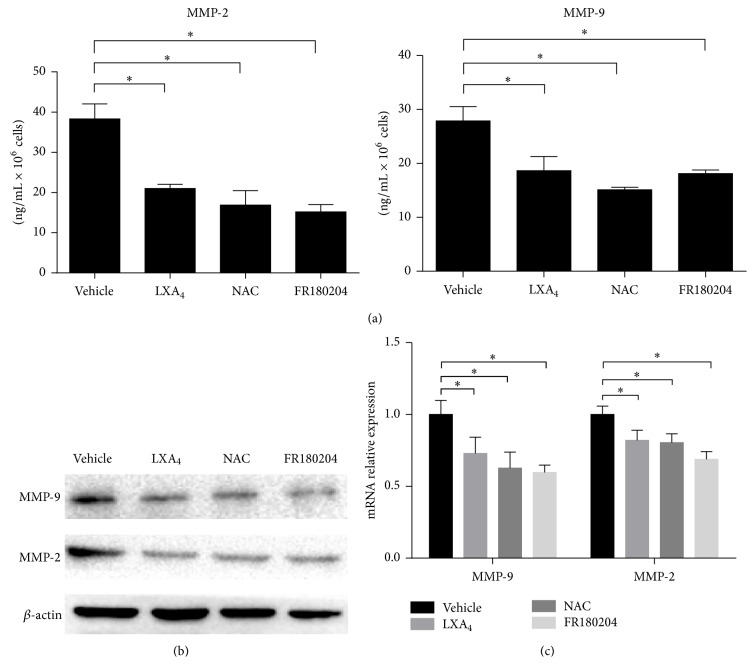
LXA_4_ downregulated MMP-9 and MMP-2 mRNA transcription. (a) Secretion of MMP-9 and MMP-2 influenced by LXA_4_, NAC, or FR180204. Cells were cultured with FBS-free medium for 24 hours and then MMP-9 and MMP-2 secreted into mediums normalized by cell number were tested by ELISA. (b) Western blot analysis of MMP-9 and MMP-2 in Panc-1 cells treated like above. (c) Transcription of MMP-9 and MMP-2 tested by RT-qPCR.  ^*∗*^ *P* < 0.05 versus vehicle control.

Our previous data demonstrated that LXA_4_ could inhibit cell invasion via the downregulation of MMP-9/MMP-2 and the suppression of ROS/ERK pathway. However, it still needed to investigate how LXA_4_ influenced the expression of MMPs. Thus we performed ELISA assay to test secreted MMPs, which showed fewer amounts MMP-9 and MMP-2 were secreted by cells treated with LXA_4_ (Figure 4(a)). At the protein level, as previous data (Figure 4(b)) have shown, MMPs were expressed to a lesser extent in the LXA_4_-treated group. Eventually, RT-qPCR demonstrated that LXA_4_ could downregulate MMP-9 and MMP-2 at the transcriptional level (Figure 4(c)).

### 3.5. LXA_4_ Reverses CoCl_2_-Induced Cell Invasion through the ROS/ERK/MMP Pathway

**Figure 5:**
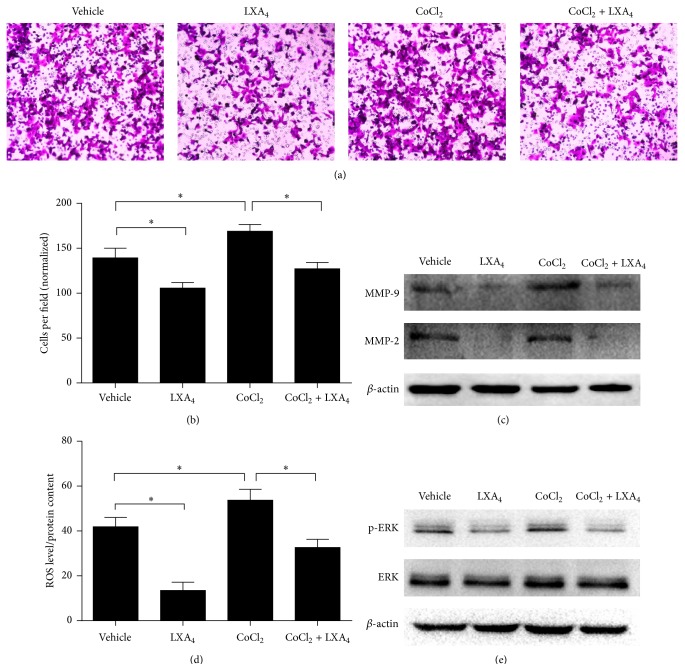
LXA_4_ reverses CoCl_2_-induced cell invasion through ROS/ERK/MMP pathway. (a) Effect of LXA_4_ on CoCl_2_-induced cell invasion. Panc-1 cells were treated with vehicle (methanol), LXA_4_, CoCl_2_ (0.15 mM), or CoCl_2_ + LXA_4_. Cell invasion assay was performed when cells had been transferred into transwell chamber for 48 hours. (b) The quantified data of (a). (c) Western blot analysis of cells treated as above. (d) Intracellular ROS determined in cells treated in (a). Cells incubated with DCF-DA for 20 min were washed with PBS three times and then lysed by RIPA lysis buffer and tested by fluorimetry at 510 nm. The absorbance was normalized by total protein. (e) Expression of activated p-ERK and total ERK detected by western blot.  ^*∗*^ *P* < 0.05 versus corresponding control.

According to our previous study [21, 22], pancreatic cancer is a type of malignancy that demonstrates poor perfusion, and consequently a hypoxic microenvironment can dramatically increase intracellular ROS which may promote cell invasion and epithelial-mesenchymal transition (EMT). To test whether hypoxia could increase MMP-9 and MMP-2 levels and whether LXA_4_ could reverse this overexpression, we added 0.15 mM CoCl_2_ to mimic the cellular hypoxic state. In cell invasion assay, after a comparison with cells that were treated with vehicle control, we found that cells treated with CoCl_2_ became more aggressive in nature. However, when they were treated with CoCl_2_ + LXA_4_, the number of cells that passed through the Matrigel decreased (Figures 5(a) and 5(b)), which suggested LXA_4_ reversed CoCl_2_-induced cell invasion. Next, the expression of MMP was measured. Cells that were treated with CoCl_2_ overexpressed MMP-9 and MMP-2, which was reversed by CoCl_2_ + LXA_4_ (Figure 5(c)). This demonstrates that LXA_4_ could reverse the CoCl_2_-induced overexpression of MMPs. Furthermore, an assay to determine intracellular ROS assay showed that CoCl_2_ upregulated intracellular ROS while LXA_4_ could attenuate that effect (Figure 5(d)). In addition, the cellular ERK pathway was activated when the cells were cultured with CoCl_2_, but it was inactivated by LXA_4_ (Figure 5(e)). These data implied that inactivation of the ROS/ERK/MMP pathway might be involved in the reversal of CoCl_2_-induced cell invasion.

## 4. Discussion

Pancreatic cancer is characterized by early invasion and metastasis, which partially account for a compromised therapeutic effect and poor outcome [23]. Therefore, it is necessary to establish new methods to control cell invasion and metastasis. In the present study, we show that LXA_4_ tends to attenuate cell invasion* in vitro*.

LXA_4_ has been described as an anti-inflammatory and proresolution small lipid mediator. Over the last few decades, several studies have reported that LXA_4_ might exert powerful anticancer effects. Here, our results demonstrate that LXA_4_ downregulates intracellular ROS to inhibit cell invasion, which is in agreement with data of previous studies on inflammation [18, 19] and endothelial cells [17]. Some studies have revealed that the LXA_4_ analog ATL acts as a nicotinamide adenine dinucleotide phosphate (NADPH) oxidase inhibitor, and thus it can block the production of intracellular ROS [17, 19]. In addition, LXA_4_ can also block neutrophil-platelet interactions; this reduces neutrophil-derived ROS, which is a characteristic of inflammation [18]. However, the results of another study contradict the aforementioned results. That study showed that LXA_4_ activates rather than blocks NADPH oxidase and COX-2 to elevate ROS production in rat aortic cells [24], which indicates that LXA_4_ may have different functions in different tissues.

ROS were originally regarded as promoters of cancer because of their role in tumor initiation, promotion, progression, and tissue destruction [25]. However, accumulating evidence indicates that ROS may play dual roles in cancer in a dose-dependent manner [7, 8]. On the one hand, mild intracellular ROS orchestrates various cell signals to promote cancer advancement, and therefore the suppression of ROS can attenuate cancer progression, including invasion. Our data show that the intracellular ROS inhibited by LXA_4_ or NAC reduce cell invasion and thus support this perspective, as in our previous study, where we illustrated that the depletion of H_2_O_2_ by catalase limits pancreatic cell invasion [22]. On the other hand, extremely high levels of ROS, which are usually induced by radiation therapy or chemotherapeutic agents, destroy almost all cellular components, which then triggers cell death. The present study is not concerned with radiation and other therapeutic agents, and thus the intracellular ROS level is not so high as to limit cancer progression; hence, the scavenging of ROS by LXA_4_ induces anticancer effects.

Invasion is widely accepted as a hallmark of cancer [26], especially in pancreatic cancer. Studies have been conducted in this field for several decades, but invasion is still responsible for the poor outcome of patients with pancreatic cancer. In recent years, studies that have focused on the tumor microenvironment revealed that a remodeled tumor extracellular matrix (ECM), which is affected by cancer cells and stroma, facilitates cancer cell invasion [25, 27, 28]. MMPs secreted by cancer cells play a key role in the degradation of the ECM, which weakens the natural barrier and inhibits cell invasion [29]. However, MMPs are regulated by different cellular signals. Several studies have demonstrated that mitogen-activated protein kinase (MAPK) pathways, especially ERK, regulate MMP expression [12, 13, 30–32]. In fact, most patients with pancreatic cancer carry mutational activation of the KRAS oncogene [23] which partially accounts for a dramatically activated ERK pathway, overexpression of MMPs, and obvious invasive potential [20]. Additionally, an overactivated ERK pathway in cancer may also be regulated by ROS [33]. In our study, through a comparison of intracellular ROS and ERK activation between the NAC- and the FR180204-treated groups, we can conclude that ROS acts upstream of ERK. This result is in accordance with that of other studies discussed above. Furthermore, our data also elucidate that the inhibition of the ROS/ERK pathway by LXA_4_ efficiently downregulates the expression of MMP-9 and MMP-2, which attenuates cell invasion. Finally, we confirmed that the inhibitory effect of LXA_4_ on the expression of MMPs is implemented at the transcriptional level.

Poor perfusion is another characteristic of pancreatic cancer [23], which typically is associated with a hypoxic microenvironment. Hypoxia promotes pancreatic cancer progression through various means including the enhancement of cell invasion [21, 34]. In the last part of our study, we treated the cells with CoCl_2_ to mimic a hypoxic environment. Our results show that ROS production dramatically increases with hypoxia and that consequent ERK pathway activation leads to the overexpression of MMP-9 and MMP-2, which promotes cell invasion. Encouragingly, the protective effect of LXA_4_ exists even in this hypoxic model, which indicates that LXA_4_ is more likely to be effective against pancreatic cancer* in vivo*.

In summary, our present study showed that the endogenous AA metabolite LXA_4_ could attenuate pancreatic cancer cell invasion via the inhibition of the ROS/ERK/MMP pathway. Our data also revealed that in a CoCl_2_-induced hypoxic model cancer cells tended to upregulate the ROS/ERK/MMP pathway to obtain aggressive, invasive behavior and that this effect could be reversed by LXA_4_. This implies that LXA_4_ may be a novel agent that targets the ROS/ERK/MMP pathway to prevent or control cancer cell invasion.

## 5. Conclusion

Our work demonstrates that LXA_4_ attenuates cell invasion in pancreatic cancer by suppression of the ROS/ERK pathway and consequent MMP-9/MMP-2 transcription not only in a pancreatic cancer cell line but also in a CoCl_2_-induced model of hypoxia. This suggests that LXA_4_ may be a novel agent that targets the ROS/ERK/MMP pathway to prevent or control cancer cell invasion.

## Supplementary Material

Supplementary Table: The sequences of primers for RT-qPCR.
