# Supplementary material for: Lipoxin A4 Attenuates Cell Invasion by Inhibiting ROS/ERK/MMP Pathway in Pancreatic Cancer
Source: Oxid Med Cell Longev. 2015 Nov 16;2016:6815727. doi: 10.1155/2016/6815727 (PMC4663743; doi:10.1155/2016/6815727)
Supplement: Supplementary file 1 — Supplementary Table: The sequences of primers for RT-qPCR. [file 6815727.f1.pdf]

Supplementary Table 1 Primers used in RT-qPCR

Product	Forward Primer (5’-3’)	Reverse Primer (5’-3’)
MMP-9	GCAATGCTGATGGGAAACCC	AGAAGCCGAAGAGCTTGTCC
MMP-2	ACCAGCTGGCCTAGTGATGA	CTGGGGCAGTCCAAAGAACT
β-actin	AGCGAGCATCCCCCAAAGTT	GGGCACGAAGGCTCATCATT
